# Trauma in Pregnancy and Its Consequences in Kermanshah, Iran From 2007 to 2010

**DOI:** 10.5539/gjhs.v7n2p304

**Published:** 2014-12-02

**Authors:** Maryam Zangene, Behzad Ebrahimi, Farid Najafi

**Affiliations:** 1 Department Obstetrics and Gynecology, Medical School, Kermanshah University of Medical Sciences, Kermanshah, Iran; 2 Kermanshah University of Medical Sciences, Kermanshah, Iran; 3 Epidemiology Department, School of Population Health, Kermanshah University of Medical Sciences, Kermanshah, Iran

**Keywords:** trauma, pregnancy, fatality, Kermanshah

## Abstract

**Objective:**

Nowadays, with decreased mortality of pregnant women by obstetrical causes, trauma has become a leading cause of morbidity and mortality in pregnant women. This study was carried out to determine the frequency of trauma in pregnancy and related causes and selected consequences in pregnant women of Kermanshah, Iran from 2007 to 2010.

**Methods:**

In this descriptive-analytical study, all pregnant women who suffered trauma and were admitted to Imam Reza, Taleghani, and Motazedi hospitals located in Kermanshah from 2007-2010 were studied. Sampling was done by census method and medical records of all eligible patients were studied. Data analysis was done by the SPSS software for Windows 9ver. 16.0).

**Results:**

There were 102 cases of trauma in pregnancy registered in this time period. Mean age of the cases was 26 years. Most cases (43%) were in their third trimester of pregnancy upon admission. Most trauma cases were of blunt traumas (68%). In 68 cases (66.67%), trauma resulted in maternal injury (independent of pregnancy) and 13 cases (12.75%) resulted in obstetrical or fetal injuries. Maternal injuries showed significant difference (P= 0.02) in different years. Motor vehicle accidents with a frequency of 47% were the most common cause of trauma.

**Conclusion:**

Trauma in pregnancy can be a leading cause of injury and fatality in mother and fetus. The most common type of injury was motor vehicle accidents. Therefore, any strategy that can decrease the rate of motor vehicle accident in a community can decrease mortalities of women (even pregnant or non-pregnant).

## 1. Introduction

During the last century, with decreased mortality of pregnant women by obstetrical causes, the role of non-obstetrical causes in mortality of this group of women has increased. Trauma has become a leading cause in mortality and complications in such patients (Kingston et al., 2003). Trauma occurs in about 6-7% of pregnancies and has unfavorable outcomes in mother, her fetus, neonate and then infant (El Kady, 2007). About 0.2% of pregnant women require hospital admission due to traum) (Mattox & Goetzl, 2005; Ali et al., 2007). Based on some studies, trauma is the mortality cause in 46% of pregnant women and is the responsible cause in 5% of fetal demise cases (Kissinger et al., 1991; Weiss et al., 2001). There is no definite statistics about trauma prevalence in Iran. Most trauma cases during pregnancy are minor traumas with good prognosis for mother and fetus (Grossman, 2004). Trauma mechanism can be divided into penetrating or blunt trauma. Blunt traumas are more common than penetrating traumas (Petrone et al., 2011). Prominent causes of trauma during pregnancy include motor vehicle accidents (the most common), falling, assaults, homicide, domestic violence, and penetrating injuries (bullet and stab wounds) (Oxford & Ludmir, 2009). As expected, the prevalence of these injuries is dependent on cultural and social characteristics of a society. For example, in the US motor vehicle accident trauma is the leading cause of traumatic death in pregnant women followed by violence and assault (Brown, 2009).

Complications that threaten pregnant women following trauma are injury or death of the mother, shock, internal hemorrhage, intrauterine fetal demise, direct injury to the fetus, spontaneous abortion, premature rupture of fetal membranes, preterm labor, cesarean section, placental abruption, and uterine rupture (Mirza et al., 2010). Even though it is anticipated that the complications of trauma be proportional to the trauma severity and mechanism, minor traumas can result in complications such as preterm labor, placental abruption, maternal-fetal hemorrhages, and fetal death (Weiss et al., 2001; Chames, & Pearlman, 2008).

Health status in pregnant women is an index of general health in a community. Trauma is a main cause of morbidity and mortality in this group. As stated previously, prevalence and occurrence of trauma and its complications is dependent on cultural and social characteristics of every region. Hence, with objective of determining the frequency of trauma causes and some of its consequences, and, this study was carried out, which can also serve as an introduction for further studies. The results obtained can be used for preventive cultural and social measures in the future.

## 2. Methods

### 2.1 Samples

In this descriptive-analytical study, all pregnant women who suffered trauma and were admitted to Imam Reza, Taleghani, and Motazedi hospitals located in Kermanshah, Iran from 2007-2010 were studied. Sampling was done by census method and medical records of all eligible patients were studied.

### 2.2 Data Collection

The required data including maternal age, gestational age, type and cause of trauma, type of inflicted injury to mother and fetus were documented in a checklist.

### 2.3 Statistical Analysis

The gathered data were analyzed using the SPSS software for Windows (ver. 16.0). Mean and standard deviation (SD) were calculated for nominal data. Frequency and percentage were calculated for categorical data. The Chi-squared test and Fisher’s exact test were used to compare categorical data. To determine collective incidence of maternal and fetal complications of trauma, the frequency of these complications was divided by the total number of incidents and 95% confidence interval was calculated for these complications.

## 3. Results

Of 102 admitted patients with trauma during pregnancy, mean age of the mothers was 26 years and 58% were nulliparous and 42% were multiparous. Ninety-nine subjects were housewives, two were university students, and one case had clerical job. The trauma cases were more common in the third trimester (43.33%) compared to the first (23.53%) and second trimester (33.14%). Blunt traumas (69 cases, 68%) were two times more common than penetrating traumas (33 subjects, 32%). The difference between proportions of penetrating traumas at different years was not significant, but difference between proportions of injuries at different years was significant (P=0.02). Fetal injury was not significantly different between nulliparous (13.6%) and multiparous (11.6%) women (Table 1).

**Table 1:** Frequency distribution of traumas during pregnancy and resultant injuries in Kermanshah, Iran, 2007-2010

		Number of trauma	Type	Frequency (%)	P value	Maternal injury			Fetal injury		

						Injury	Frequency (%)	P value	Injury	Frequency (%)	P value
**Hospital**	Imam Reza	45	Penetrating	21 (47%)	Χ^2^= 8.73 df= 2 P= 0.013	Yes	31 (69%)	Χ^2^= 16.5 df= 2 P= 0.0003	Yes	9 (20%)	†-
		
			Blunt	24 (53%)		No	14 (31%)		No	36 (80%)	
		
	Taleghani	39	Penetrating	10 (26%)		Yes	32 (82%)		Yes	2 (5%)	
		
			Blunt	29 (74%)		No	7 (18%)		No	37 (95%)	
		
	Motazedi	18	Penetrating	2 (11%)		Yes	5 (72%)		Yes	2 (11%)	
		
			Blunt	16 (89%)		No	13 (28%)		No	16 (89%)	

**Year**	2007	12	Penetrating	6 (50%)	Χ^2^= 2.43 df= 3 P= 0.49	Yes	10 (83%)	Χ^2^= 9.83 df= 3 P= 0.02	Yes	3 (25%)	-
		
			Blunt	6 (50%)		No	2 (17%)		No	9 (75%)	
		
	2008	24	Penetrating	6 (25%)		Yes	10 (42%)		Yes	3 (12.5%)	
		
			Blunt	18 (75%)		No	14 (58%)		No	21 (87.5%)	
		
	2009	11	Penetrating	4 (36%)		Yes	7 (64%)		Yes	0	
		
			Blunt	7 (64%)		No	4 (36%)		No	11 (100%)	
		
	2010	55	Penetrating	17 (31%)		Yes	41 (75%)		Yes	7 (13%)	
		
			Blunt	38 (69%)		No	14 (25%)		No	48 (87%)	

**Nulliparous**		43	Penetrating	14 (23%)	Χ^2^= 0.0014 P= 0.97	Yes	26 (60%)	Χ^2^= 1.29 P= 0.26	Yes	6 (14%)	Χ^2^= 0.098 P= 0.75
		
			Blunt	29 (67%)		No	17 (40%)		No	37 (86%)	
		
**Multiparous**		59	Penetrating	19 (32%)		Yes	42 (71%)		Yes	7 (12%)	
		
			Blunt	40 (68%)		No	17 (29%)		No	52 (88%)	

**Occupation**¶	Housewife	99	Penetrating	31 (31%)	P= 0.488	Yes	66 (67%)	P= 0.999	Yes	12 (12%)	P= 0.677
		
			Blunt	68 (69%)		No	33 (33%)		No	87 (88%)	
		
	Student	1	Penetrating	0		Yes	0		Yes	0	
		
			Blunt	1 (100%)		No	1 (100%)		No	1 (100%)	
		
	Clerk	2	Penetrating	2 (100%)		Yes	2 (100%)		Yes	1 (50%)	
		
			Blunt	0		No	0		No	1 (50%)	

† No P value report means that they did not have the Chi-squared pre-assumptions.

The most common mechanism of trauma was motor vehicle accidents (47%). Seventy-four cases (73%) were unintentional trauma and 18 cases (17%) were intentional and for other cases, no report was recorded. Distribution of these cases was different according to mechanism of trauma and 93% was due to fighting (Table 2).

**Table 2:** Frequency and trauma intention (intentional, unintentional, unknown) according to trauma mechanism in pregnant women, Kermanshah, Iran, 2007-2010

Trauma type	Frequency	Intentional, frequency (%)	Unintentional, frequency (%)	Unknown, frequency (%)
Vehicle accident	48	1 (2%)	45 (94%)	2 (4%)
Falling	16	1 (4%)	19 (73%)	6 (23%)
Fighting	26	15 (94%)	1 (94%)	0
Stab wound	2	1 (50%)	0	1 (50%)
Others	10	0	9 (90%)	1 (10%)
Total	102	18 (17%)	74 (73%)	10 (10%)

**Figure 1:**
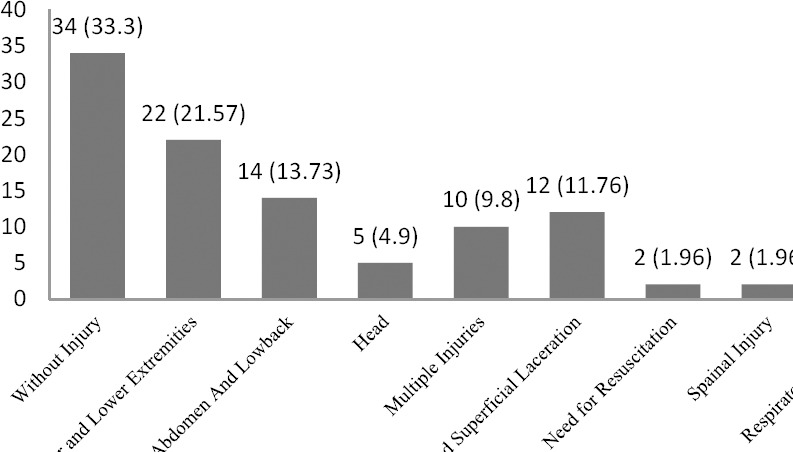
Frequency Distributionof traumaonmaternaldamages, Kermanshah, Iran, 2007-2010

Of all registered cases, 68 cases (66.67%) resulted in maternal injury (independent of pregnancy) and 13 cases (12.75%) resulted in fetal injuries or obstetrical-related injuries. Of maternal injuries, extremity injuries followed by abdominal and low back injuries were the most common ones (Figure 1).

Of injuries related to the fetus or pregnancy itself, the most common ones were placental abruption and labor onset. Distribution of these traumas was different according to trauma type (Table 3) and trauma mechanism (Table 4).

**Table 3:** Frequency of fetal- or obstetrical-related traumas according to trauma type

Fetal injury type	Frequency	Penetrating, frequency (%)	Blunt, frequency (%)
Without fetal complication	77	22 (29%)	55 (71%)
Placental abruption	6	2 (33%)	4 (67%)
Labor onset	6	3 (50%)	3 (50%)
Premature rupture of fetal membranes	5	2 (40%)	3 960%)
Abortion	4	2 (50%0	2 (50%)
Stillbirth	3	2 (67%)	1 (33%)
Uterine rupture	1	0	1 (100%)

Total	102	33 (32%)	69 (68%)

**Table 4:** Frequency of fetal- or obstetrical-related traumas according to trauma mechanism

	Placental abruption	Labor onset	Premature rupture of membranes	Abortion	Stillbirth	Uterine rupture	Without fetal complication	Total
Vehicle accident	5 (83.33%)	2 (33.33%)	4 (080%)	3 (75%)	1 (33.33%)	0	33 (42.84%)	48 (47.05%)
Fighting	0	1 (16.67%)	0	0	0	0	15 (19.48%)	16 (15.68%)
Falling	1 (16.67%)	2 (33.33%)	1 (20%)	0	1 (33.33%)	1 (100%)	20 (25.98%)	26 (25.5%)
Stab wound	0	0	0	0	0	0	1 (1.3%)	1 (0.98%)
Others	0	1 916.67%)	0	1 (25%0	1 (33.33%)	0	8 (10.39%)	11 (10.78%)

Total	6	6	5	4	3	1	77	102

Maternal/fetal death was reported in three cases (2.9%). One case (0.98%) was due to fighting between mother and her husband and stab wound to the abdomen which resulted in rapid death of the mother and fetus. Two cases were due to motor vehicle accidents which resulted in death of the mothers and fetuses at the scene (1.96%).

Due to small numbers, occupation was regarded as housewife or non-housewife and P value was calculated using the Fisher’s exact test.

## 4. Discussion

The obtained results herein show that blunt and penetrating traumas respectively constituted 68% and 32% of traumas during pregnancy in our society. Maternal injury of different trauma cases was 67% and fetal injury was 13%. The most common causes of maternal and fetal injuries were motor vehicle accidents, falling, fighting with husband and maternal stab wound and other traumas (insect bite, explosion, and pollution).

Mean age of pregnant women in this study was 26 years, but in Aboutanos study, this figure is 23.8 years (Aboutanos et al., 2008). It seems that high percentage of traumas due to motor vehicle accidents to be a major cause for higher mean age of mothers in this study.

When comparing the current results with another study performed in the US, the main causes of trauma in American study were motor vehicle accident (49%), falling (25%), sexual assault (18%), and bullet injuries (4%). There were limited cases of fighting between mother and her husband and this item was not calculated. In our study the causes of trauma in order of prevalence were motor vehicle accident (47%), falling (26%), fighting with husband (16%), other traumas (insect bite, explosion, pollution) 11% and stab wound 1%. High number of traumas due to motor vehicle accident is common in both studies, but fighting with husband was more common in our study. Also, sexual assault was common in American study, but this type of injury was not recorded here (El Kady, 2007).

The prevalence of penetrating trauma was 32% and blunt trauma was 68%, while in Aniuliene study the prevalence of penetrating and blunt traumas was 16% and 84%, respectively (Aniuliene et al., 2006). This reflects higher prevalence of penetrating trauma in our study.

The most common complications of trauma in our study were placental abruption, labor onset (preterm labor), premature rupture of membranes, abortion, stillbirth, and uterine rupture. In a similar study in the US by Weiss et al. (2001) in 2001 the leading cause of maternal and fetal injury and death was motor vehicle accidents followed by bullet injury and falling. They reported maternal mortality as 11% and the most common obstetrical injury was placental injuries including placental abruption (42%).

In another report from Finland, the medical records of 35 pregnant women who suffered trauma were reviewed. One mother and her fetus died at the scene of motor vehicle accident due to spinal column injury and uterine rupture. In 4 other fetal death cases, death occurred upon admission to hospital and placental abruption was the cause in all four cases (Chames & Pearlman, 2008).

In another study in Sweden in 2008 (Kvarnstrand et al., 2008), motor vehicle accident in pregnancy was a major cause of maternal and fetal fatalities and maternal death following such trauma was three times more common than in fetuses.

In our study, the most prevalent traumas in mother in order were extremity traumas (21.5%), abdominal and low back pain (13.7%), wounds and lacerations (11.7%), multiple trauma (9.8%), head trauma (4.9%), spinal injury (1.9%), and respiratory problems (0.98%). Of 102 reviewed medical records, maternal and fetal mortality was three cases. One fatality (0.98%) was due to fighting with husband and stab wound to the mother’s abdomen which resulted in rapid death of the mother as well as her fetus. Two fatalities (1.96%) were due to motor vehicle accident which resulted in death of the mothers and fetuses at the scene.

In our study, motor vehicle accident was the most common cause of pregnant women mortality due to trauma followed by fighting with husband and stab wound by husband. In similar studies, mortality due to motor vehicle accidents was the leading cause, while mortality due to fighting with husband was trivial.

## 5. Conclusion

Considering the current findings and similar reports, trauma during pregnancy can be a leading cause in maternal/fetal injury/death. The most common injury was due to motor vehicle accident. Therefore, any strategy that can decrease the rate of motor vehicle accident in a community can decrease mortalities of women (even pregnant or non-pregnant). Planning is essential to change the rules of accident victims in hospital.

This study included pregnant women trauma cases which necessitated presentation to hospital not those minor cases in which pregnant women did not sought medical attention from a hospital service. It is likely that some women did not mention the real cause of trauma as domestic violence, and instead reported other causes such as falling as the cause of inflicted trauma. Hence, explicit judgment about various causes of trauma is somehow difficult.

In investigating the consequences of trauma, we only studied direct and short-term consequences which were documented in the medical records of the pregnant women. We believe that with more comprehensive studies and implementing cohort studies, psychological as well as more long-term consequences of trauma in pregnant women will be discovered.
